# Primary Care Records and Population Prevalence of Chronic Insomnia: Do They Match?

**DOI:** 10.3390/healthcare13233152

**Published:** 2025-12-03

**Authors:** Jesús Pujol Salud, Cristina García-Serrano, Manuel de Entrambasaguas, Maria Malla Montagut, Javier Martínez Redondo

**Affiliations:** 1 Balaguer Primary Health Care Center, Institut Català de la Salut (ICS), 08007 Barcelona, Spain; cgarcias.lleida.ics@gencat.cat (C.G.-S.); mmalle.lleida.ics@gencat.cat (M.M.M.); jmartinez.lleida.ics@gencat.cat (J.M.R.); 2 Multiprofessional Teaching Unit for Family and Community Care, Institut Català de la Salut (ICS), 08007 Barcelona, Spain; 3 Medicine Department, University of Lleida, 25198 Lleida, Spain; 4 Multidisciplinary Research Group on Therapeutics and Interventions in Primary Care (RETICAP Group), Fundació Institut Universitari per a la Recerca a l’Atenció Primària de Salut Jordi Gol i Gurina (IDIAPJGol), Gran Via de les Corts Catalanes, 587, 08007 Barcelona, Spain; 5 Clinical Neurophisiology, Clinical University Hospital of Valencia, INCLIVA Biomedical Research Institute, 46010 Valencia, Spain; entrambasaguas_man@gva.es

**Keywords:** sleep initiation and maintenance disorders, primary health care, big data, surveys and questionnaires

## Abstract

**Background/Objectives:** Chronic insomnia is a prevalent condition with important health implications. However, its recognition in clinical practice is often limited. This study evaluated the alignment between population-based estimates of chronic insomnia and the prevalence recorded in primary care, assessing the diagnostic reliability of healthcare services. Design: We conducted a comparative cross-sectional analysis using two independent data sources: (1) the Epidemiology Study of Insomnia in Spain (EPINSOM) survey, which applied the International Classification of Sleep Disorders, Third Edition (ICSD-3), criteria to a representative sample of the Spanish adult population (*n* = 2243; Catalonia subsample *n* = 363) and (2) the SIDIAP Electronic Health Record (EHR) database, comprising anonymized primary care data from adults in Catalonia (*N* = 4,131,754). **Methods:** Prevalence estimates of insomnia symptoms, chronic insomnia syndrome, and chronic insomnia disorder were extracted from the EPINSOM survey and compared with ICD-10-coded insomnia diagnoses in the SIDIAP. Pairwise comparisons of proportions were conducted using z-tests for independent samples. For transparency, 95% confidence intervals (CI) were calculated using the Wilson method. **Results:** In Catalonia, the population-based prevalence of chronic insomnia disorder was 13.6% (95% CI: 10.1–17.1), whereas the prevalence of coded insomnia diagnoses in primary care was significantly lower at 5.1% (95% CI: 5.08–5.12). Among adults aged ≥55 years, the prevalence estimates were more closely aligned between the survey (18.2%) and SIDIAP records (18.5%). The survey-derived prevalence of insomnia symptoms reached 41.39% (95% CI: 39.2–43.6), highlighting substantial underrecognition in clinical practice. **Conclusions:** Our findings indicate that primary care insomnia diagnoses substantially underestimate insomnia prevalence compared with ICSD-3-based population estimates, particularly in younger adults. While diagnoses appear to be more like to prevalence in older adults (>55 years), this discrepancy highlights the need for improved identification and management of insomnia in primary care settings, especially among younger populations who may be underdiagnosed.

## 1. Introduction

Insomnia is one of the most common sleep disorders in the general population, with epidemiological studies estimating that 6–15% of adults meet the diagnostic criteria for an insomnia disorder, depending on the definitions and populations studied. Prevalence estimates vary between 5% and 20% depending on the diagnostic criteria used and the population studied [1,2].

Beyond its prevalence, insomnia is critically relevant because of its strong association with other medical and psychiatric conditions [3,4]. Common comorbidities include respiratory diseases such as chronic obstructive pulmonary disease (COPD), asthma, cardiovascular diseases, diabetes mellitus, rheumatic disorders, and chronic pain syndromes. Anxiety and depression often coexist with insomnia [5], complicating its management and increasing the burden on the healthcare system. Substance use, including tobacco and alcohol use, is also associated with a higher incidence of insomnia [6].

Because many of these comorbidities are managed in the community, primary care is pivotal for the early identification and treatment of insomnia [7]. A primary care approach allows healthcare providers to use a more holistic framework, ensuring that insomnia is not overlooked and that its management is coordinated with the treatment of co-occurring conditions. Such integration can improve patient outcomes and healthcare system efficiency by comprehensively addressing interconnected health issues [8].

Despite the availability of clinical practice guidelines [9] and increasing awareness of evidence-based treatments, insomnia often remains underdetected in primary care. Barriers include limited consultation time, low prioritization of sleep complaints, variability in training, and absence of systematic approaches for assessing sleep. This gap between the population burden and clinical recognition underscores the need to understand how well primary care diagnoses reflect the true prevalence of insomnia in the community. Moreover, the implementation of these guidelines often varies across countries [10].

In Spain, training on insomnia within family medicine specialization programs is often inadequate or nonexistent, resulting in variability in the knowledge and skills of primary care professionals regarding diagnosis and management [11]. As primary care is the first point of contact for many patients with insomnia, this lack of specialized training may hinder appropriate detection and treatment, thus perpetuating the problem [12].

Therefore, the present study compared two independent data sources: the EPINSOM population survey, which estimated insomnia prevalence in a representative sample of adults in Spain using the International Classification of Sleep Disorders, Third Edition (ICSD-3), criteria and the SIDIAP electronic health record (EHR) database, which contains anonymized primary care data for approximately 80% of the population of Catalonia. Catalonia was selected because it provides one of the most comprehensive and validated primary care databases in Europe, enabling a robust epidemiological assessment of recorded diagnoses at the population level.

The hypothesis of the present study is that there will be significant discrepancies between the prevalence of chronic insomnia disorder estimated from population-based surveys and that recorded in primary care electronic health records. We based our hypothesis on the premise that chronic insomnia prevalence provided by diagnostic records in primary care clinical histories differs from the prevalence rates in the general population. This situation indicates that primary health services are unreliable for diagnosing this specific health issue. Therefore, this study aimed to investigate the correlation between the prevalence of insomnia calculated from population surveys and the proportion of insomnia diagnoses from a large clinical data repository.

## 2. Materials and Methods

The primary objectives of this study were to (1) compare the prevalence of chronic insomnia disorder estimated from a population-based survey with the prevalence recorded in primary care electronic health records; (2) examine differences in these prevalence estimates by age and sex; and (3) discuss potential factors contributing to any observed discrepancies, particularly those related to diagnostic practices in primary care.

A comparative analysis was conducted using two primary data sources: (1) results from a population-based survey and (2) data extracted from primary care electronic health records. This approach was used to evaluate the reliability and effectiveness of health services for the detection and management of chronic insomnia.

### 2.1. Population-Based Survey

This study was conducted as a comparative secondary data analysis using two independent datasets from a similar period: the EPINSOM population-based survey (2018–2019) and the SIDIAP primary care electronic health record (EHR) database (snapshot as of 31 December 2018). The objective was to compare population-based prevalence estimates of insomnia with primary-care-recorded diagnoses and to assess the degree of alignment between the two sources. The temporal proximity of data collection allowed for a meaningful comparison of prevalence estimates across datasets.

EPINSOM was a cross-sectional telephone survey conducted in 2018 (*n* = 1500) and 2019 (*n* = 743), providing a total sample of 2243 adults representative of the Spanish population. A subsample of 363 respondents residing in Catalonia was included in this analysis. Sampling followed a stratified random design based on sex, age, geographical region, and municipality size using distributions from the National Statistics Institute. Adults aged 18 years were considered eligible. The exclusion criteria included nightshift work and communication limitations that prevented participation.

Prior to data collection, interviewers underwent specific training on sleep disorders and study protocols, and the interviewers underwent a one-day (8 h) training session led by experienced sleep researchers. The training covered the following topics.

- Sleep Disorders Overview: A general introduction to sleep disorders, with a focus on insomnia, its subtypes, and its impact on health and quality of life.
- ICSD-3 Diagnostic Criteria: Detailed instructions on the International Classification of Sleep Disorders, Third Edition (ICSD-3), diagnostic criteria for chronic insomnia disorder, including inclusion and exclusion criteria. Emphasis was placed on understanding the nuances of each criterion and on how to apply them consistently.
- Survey Instrument Administration: Hands-on practice with the survey instrument, including instruction on how to ask questions in a standardized manner, how to probe for clarification when necessary, and how to record responses accurately.
- Ethical Considerations: Training on maintaining participant confidentiality, obtaining informed consent, and handling sensitive information.
- Study Protocols and Data Security: A review of the protocols for data collection, storage, and security.

The training included a combination of lectures, interactive discussions, role-playing exercises, and practical interviews. Interviewers were provided with a training manual that contained all relevant information. A quiz was administered at the end of the training to ensure the comprehension of the material. Only interviewers who passed the quiz were allowed to participate in the data collection.

The survey instrument was designed based on the International Classification of Sleep Disorders, Third Edition (ICSD-3), diagnostic criteria. Questions addressed the following topics.

- Nocturnal and daytime symptoms.
- Frequency and duration of symptoms (almost three nights per week for three months a year).
- Inadequate sleep opportunity or environment and presence of other sleep disorders (exclusion criteria).
- Medication use for sleep.

A stepwise approach was used to ensure accurate prevalence estimation, starting from basic nocturnal symptoms and progressively incorporating the ICSD-3 inclusion and exclusion criteria and relevant notes. Logical language commands were used to describe each diagnostic criterion and note in the survey algorithm. The raw data were weighted to extrapolate the findings to the broader Spanish adult population, accounting for the inverse probability of selection.

For this study, information from respondents residing in Catalonia, an autonomous community in Spain, was processed.

### 2.2. Primary Care Electronic Health Record Data (SIDIAP)

SIDIAP contains anonymized EHR data from approximately 80% of the Catalan population. Adults aged ≥ 18 years with an active diagnosis of insomnia were included. Chronic insomnia was defined as an active ICD-10 insomnia code (F51.0 or G47.0) recorded during the three-month period preceding December 31, 2018, consistent with the ICSD-3 chronicity criterion. The SIDIAP automatically consolidates repeated or overlapping diagnostic entries within each patient’s longitudinal record; only active diagnoses were considered, and historical or inactive codes were excluded from the analysis.

To minimize diagnostic misclassification, individuals with severe psychiatric conditions (such as schizophrenia or bipolar disorder), neurodegenerative disorders (including Alzheimer’s disease and Lewy body dementia), fibromyalgia, restless legs syndrome, or active cancer were excluded; bipolar depressive disorder (F31.3, F31.4, F31.5), schizophrenia (F20.0, F20.5, F20.9), emotionally unstable personality disorder (F60.2), borderline personality disorder (F60.3), psychosis (F29), affective psychosis (F39) and intellectual disability (F79.9) were excluded. Patients were diagnosed with neurological diseases such as dementia (F03), vascular dementia (F01.9), Alzheimer’s disease (F00.9), Lewy body dementia (G31.8), restless legs syndrome (G25.8), cancer, and fibromyalgia (M79.7).

Given the use of population-level data from SIDIAP and a large, nationally representative sample from EPINSOM, no a priori sample size calculation was performed.

### 2.3. Methods for Statistical Comparison

To assess the alignment between the population-based and clinical estimates of the prevalence of chronic insomnia, we conducted pairwise comparisons of proportions using the z-test for two independent samples. Specifically, we compared the prevalence of Chronic Insomnia Disorder as identified in the EPINSOM population-based survey (Catalonia subsample, *n* = 363) with the prevalence derived from the SIDIAP primary care electronic health records (*N* = 4,131,754).

Comparisons were stratified by age (18–34, 35–54, and ≥55 years) and sex (male and female), as well as by the total adult population. The z-test was used to determine whether the differences in proportions were statistically significant, with a significance threshold of *p* < 0.05.

Ninety-five percent confidence intervals were used for both data sources. These intervals were calculated using the Wilson score method, which is appropriate for large sample sizes.

To compare the prevalence of insomnia symptoms, chronic insomnia syndrome, and chronic insomnia disorder across different populations and data sources, we performed pairwise comparisons of proportions using a z-test for two independent samples. This approach allowed us to assess whether the observed differences between groups (Spain vs. Catalonia, survey vs. clinical diagnoses, and total population vs. those aged > 55 years) were statistically significant. Statistical significance was set at *p* < 0.05. Analyses were conducted using the reported prevalence rates and the available sample sizes for each source.

### 2.4. Ethical Considerations

Both data sources adhered to the ethical and legal standards for human subject research. The EPINSOM was approved by the corresponding ethics committee, and verbal informed consent was obtained and documented by trained interviewers before participation. SIDIAP data were fully anonymized prior to researcher access, ensuring strict compliance with the General Data Protection Regulation (GDPR) regarding the secondary use of health information. No identifiable data were available at any stage of the analyses.

## 3. Results

### 3.1. Prevalence of Insomnia Through a Population-Based Survey

The survey was conducted in two waves [13]. The first survey took place in June 2018 and included 1500 respondents, while the second survey was conducted in July 2019 and included 743 more respondents, for a total of 2243 respondents. The distribution according to age group and sex was 459 aged 18–34 years (51.9% women), 812 aged 35–54 years (50.5% women), and 844 aged 55 years or older (54.1% women).

The Catalonia subsample of the EPINSOM survey consisted of 363 participants: 51.79% were women, 68 were aged 18–34 years (51.2% women), 169 were aged 35–54 years (50.8% women), and 126 were aged 55 years or older (54.7% women). Of the respondents, 41.39% reported insomnia symptoms, 12.9% met the criteria for chronic insomnia syndrome, and 13.6% fulfilled the full ICSD-3 diagnostic criteria for chronic insomnia. Similarly to the national sample, the prevalence was higher among women and increased with age. Among adults aged ≥55 years, the prevalence of chronic insomnia was 18.2%.

The prevalence rates in the Spanish adult population (not performing night work) for all survey respondents and for the specific group of the population aged 55 years and older are presented in Table 1. In this table, the prevalence of insomnia symptoms, chronic insomnia syndrome and chronic insomnia disorder are also presented and compared with those of respondents residing in Catalonia.

### 3.2. Prevalence of Insomnia Through Primary Care Electronic Health Record Data (SIDIAP)

The SIDIAP database included 4,131,754 adults with valid primary care records in Catalonia. The prevalence of active ICD-10 insomnia diagnoses was 5.1% (209,386) in the overall population. Women represent a higher proportion of diagnosed cases than men, and the prevalence progressively increases with age. Among adults aged ≥ 55 years, the prevalence of coded chronic insomnia diagnosis was 18.2%, which was similar to the survey-based estimate for this age group.

The total number of women diagnosed with insomnia was 3.4%, and the total number of men was 1.9%. By age 6.3% of those aged 18–34 in primary care registers had an insomnia diagnosis (56.3% women), 5.3% of those aged 35–54 (5.3% women), and 16.3% of those aged ≥55 years, 18.2% (62.4% women).

Table 2 compares the percentages of men and women with chronic insomnia in the Spanish adult population (not performing night work), the Catalonia survey, and the SIDIAP large database.

Statistical comparisons between prevalence estimates performed using the z-test for two independent proportions showed that the prevalence of insomnia symptoms in the Catalonia population-based survey (41.39%, 95% CI: 39.2–43.6%) was significantly higher than that in SIDIAP clinical diagnoses (5.1%, 95% CI: X–Y%; Z = 47.1, *p* < 0.001).

### 3.3. Comparative Analysis

#### 3.3.1. Prevalence of Insomnia Symptoms

The prevalence of insomnia symptoms was similar between the Spanish national survey (43.4%) and the Catalonia survey (41.39%) in the total population. Among those aged > 55 years, the prevalence was slightly higher in Catalonia (45.2%) than in the national population (43.3%).

In contrast, the prevalence of insomnia symptoms based on SIDIAP clinical diagnoses in Catalonia was substantially lower for the total population (5.1%) but was notably higher among those aged > 55 years (18.2%).

#### 3.3.2. Chronic Insomnia Syndrome

The prevalence of chronic insomnia syndrome was 13.7% in Spain and 12.9% in Catalonia (survey data, total population), with a modest increase in the >55 years age group (14.5% and 14.2%, respectively).

SIDIAP clinical diagnoses in Catalonia showed a much lower prevalence in the general population (5.1%) but a higher prevalence among older adults (18.2%).

#### 3.3.3. Chronic Insomnia Disorder

The overall prevalence of chronic insomnia disorder in the SIDIAP dataset was 5.1% (95% CI: 5.08–5.12%), while the corresponding prevalence in the Catalonia survey was 13.6% (95% CI: 10.1–17.1%).

The prevalence of chronic insomnia disorder was 14% in Spain and 13.6% in Catalonia (survey data, total population), increasing to 17.9% and 18.2%, respectively, among participants aged >55 years. Again, SIDIAP clinical diagnoses in Catalonia revealed a lower prevalence in the total population (5.1%) but a higher rate in the older age group (18.2%).

#### 3.3.4. Survey vs. Clinical Diagnoses

Across the entire adult population, the prevalence of chronic insomnia disorder identified through the EPINSOM survey was substantially higher than that of insomnia diagnoses recorded in primary healthcare. This discrepancy was particularly pronounced in adults aged 18–54 years, suggesting underrecognition or underdocumentation of insomnia in primary care for younger and middle-aged adults. In contrast, the estimates were more closely aligned among adults aged ≥ 55 years, indicating improved concordance between the symptoms experienced and clinical detection in older populations.

Sex differences were consistent across both data sources. Women showed a higher prevalence of insomnia in survey responses and higher rates of recorded diagnoses in primary care, although the magnitude of sex differences was greater in the survey data.

Statistical comparisons using z-tests for independent proportions confirmed significant differences between survey-based and SIDIAP-based prevalence estimates for all age and sex subgroups, except for adults aged ≥55 years, where the observed alignment resulted in non-significant differences.

### 3.4. Summary of Key Trends

Overall, the results demonstrate the following:

1. **Marked underestimation** of chronic insomnia disorder in primary care records relative to ICSD-3–based population estimates.
2. **Consistent gender disparities were observed**, with women showing a higher prevalence across both data sources.
3. **Closer alignment** between survey and primary care estimates among older adults (≥55 years) suggests age-related differences in help-seeking behavior or diagnostic recording.
4. **Significant discrepancies** between the sources for adults under 55 years of age support the hypothesis that insomnia remains undetected in primary care.

## 4. Discussion

This study compared population-based estimates of chronic insomnia derived from the EPINSOM survey with primary-care-recorded diagnoses extracted from SIDIAP electronic health records. The results revealed a substantial discrepancy between the two sources. While nearly one in seven adults in Catalonia met the ICSD-3 criteria for chronic insomnia disorder, only 5.1% had an active insomnia diagnosis recorded in primary care. This underestimation was particularly pronounced in adults aged 18–54 years. In contrast, the alignment between sources was much closer in adults aged ≥ 55 years, among whom the survey-based and primary care prevalence estimates were nearly identical. Sex differences were consistent, with a higher prevalence in women across both datasets.

These findings suggest that self-reported insomnia symptoms and disorders are highly prevalent in both Spain and Catalonia, with similar prevalence rates in both regions. Older adults (>55 years) consistently showed higher prevalence rates, regardless of the data source. Clinical diagnoses in electronic health records (SIDIAP) substantially underestimate the prevalence of insomnia compared with population-based surveys, especially in the general population. This likely reflects underdiagnosis or differences in help-seeking behavior and diagnostic practices.

A key element in interpreting our findings was the fundamental difference between the populations represented by these two data sources. The population-based survey reflects the prevalence of insomnia symptoms and disorders in the general community, independent of healthcare utilization. In contrast, SIDIAP captures only individuals who interact with the primary care system and receive a coded diagnosis during clinical encounters. This creates a structural gap between “experienced” insomnia and “recorded” insomnia, which is driven by variations in help-seeking behavior, access to care, symptom perception, and clinicians’ diagnostic and coding practices. Younger adults and individuals with milder or intermittent symptoms are particularly likely to remain invisible in health records, whereas older adults and those with greater comorbidity or symptom severity more often seek care and are diagnosed. Therefore, the discrepancy between survey- and EHR-based prevalence estimates should not be interpreted solely as underdiagnosis but as the combined effect of behavioral, contextual, and system-level factors that shape who is represented in clinical databases. Recognizing this distinction is essential for understanding the meaning and limitations of comparing these two data sources.

Several factors may explain the underrepresentation of chronic insomnia in primary care. First, insomnia is often under-prioritized in routine consultations because of limited time and competing clinical needs. Second, primary care providers may lack formal training in sleep medicine, reducing the likelihood of systematic assessment or documentation of insomnia. Third, clinical encounters rarely include standardized screening questions for sleep difficulties, contributing to missed diagnostic opportunities. Fourth, help-seeking behavior varies across demographic groups: younger adults and individuals with mild or intermittent symptoms are less likely to seek medical assistance, whereas older adults may be more inclined to discuss sleep difficulties because of a higher comorbidity burden or greater engagement with health services. Finally, sociocultural factors, including the normalization of poor sleep and stigma surrounding mental-health-related symptoms, may reduce the likelihood of insomnia being reported in clinical settings.

The discrepancy between survey and clinical data was less pronounced in the >55 years age group, where clinical diagnoses approached survey-based estimates, which could be due to increased healthcare utilization or greater severity of symptoms leading to medical consultation in this age group. Future research directions are also highlighted to increase awareness of the illness.

Our findings align with previous epidemiological studies in Spain and Europe, showing that insomnia is common in the general population but often remains undiagnosed in primary care [14,15]. Prior research has consistently highlighted the gap between community prevalence and clinical detection, particularly in younger adults and women. Studies from other European countries have similarly reported low coding rates of insomnia in electronic health records despite the substantial symptom burden. The observed concordance in the ≥55 age group is consistent with the literature, suggesting that older adults are more likely to seek care for sleep problems and that primary care physicians may be more attentive to sleep-related concerns in this population than in younger populations. Overall, our results reinforce the existing evidence that insomnia remains under-recognized in clinical practice in European primary care systems.

The fact that the setting of the study is radically different with respect to the general population served by primary care services is a serious limitation of this study; however, this methodology has been useful in supporting our hypothesis. In addition, the identification of chronic insomnia in the SIDIAP database is subject to limitations, particularly regarding the temporal criteria used to define chronicity. Specifically, the requirement of a 3-month duration may lack consistency, as a single diagnostic code recorded at any point within the 3-month window prior to 31 December 2018 could represent an isolated clinical event rather than a sustained condition. Moreover, the SIDIAP database does not provide access to clinician notes, which restricts our ability to validate the diagnostic context or to assess symptom persistence. To minimize this, we believe that a good strategy is to use the same timeframe for the study (2018).

We cannot move on to the next paragraph without commenting on the risk involved in assuming the method used by the primary care professionals. When insomnia is diagnosed step by step according to the ICSD-3 criteria and notes [13], it is difficult to find recent literature that supports the idea that family doctors apply the same level of rigor when diagnosing insomnia as they do for conditions like diabetes or COPD. Likewise, we lack evidence to argue otherwise [7,11,12]. Therefore, one of the future directions derived from the reflection carried out by the authors through the writing of this study is the realization of a study on the characterization of the diagnosis of insomnia in primary care consultations based on the same instrument designed to carry out the population survey.

Although the COVID-19 pandemic [15,16] occurred after the period our data covers, it is crucial to recognize that it may have reshaped the epidemiological landscape of insomnia in ways not captured by our study. Emerging evidence indicates significant shifts in sleep patterns, stress levels, and help-seeking behaviors during and after the pandemic [17]. Instead of interpreting our findings through this lens, we now view the pandemic primarily as a rationale for future research [18,19]. Updated population-based surveys and analyses of post-2020 electronic health records are necessary to determine whether the discrepancies observed between community prevalence and primary care diagnoses have widened, narrowed, or changed in nature. Consequently, we have reframed this issue as a forward-looking research priority rather than an interpretative element of the current results. Our data may represent an intermediate stage in this evolving landscape, and further research is essential to fully understand the pandemic’s lasting impact on insomnia prevalence and its associated risk factors. Grasping these temporal dynamics is vital for developing targeted public health strategies and ensuring equitable access to effective sleep care in the post-pandemic era.

Finally, characterizing how insomnia is diagnosed, coded, and managed in primary care remains an important area for future research. A deeper understanding of coding practices, clinical decision-making, and the use of non-pharmacological treatments, such as cognitive-behavioral therapy for insomnia (CBT-I), may contribute to improving the quality of care for patients with chronic insomnia.

## 5. Conclusions

This study demonstrated a substantial discrepancy between population-based estimates of chronic insomnia and the prevalence of insomnia diagnoses recorded in primary healthcare. Although nearly one in seven adults in Catalonia met the ICSD-3 criteria for chronic insomnia disorder according to the EPINSOM survey, only 5.1% had an active insomnia diagnosis documented in the SIDIAP electronic health record. This underestimation was most pronounced among adults aged 18–54 years, whereas the discrepancy, although reduced, persisted among adults aged ≥55 years. These findings highlight the limited visibility of insomnia in routine clinical practice across all adult age groups and underscore the need for improved detection, diagnostic assessment, and documentation in primary care settings.

Future research should focus on understanding the diagnostic pathways that lead to the recognition or omission of insomnia in primary care, including the roles of clinical training, coding practices, and help-seeking behavior. Replicating this analysis using post-pandemic data is important to determine whether changes in sleep patterns and healthcare utilization have modified the gap between community prevalence and recorded diagnosis. In addition, prospective validation studies comparing EHR-based diagnoses with structured clinical assessments would help clarify the accuracy of insomnia coding in primary care settings.

## Figures and Tables

**Table 1:** Percentages of insomnia symptoms, chronic insomnia syndrome, and chronic insomnia disorders in the survey compared with SIDIAP insomnia diagnostics by age group.

	Spain Population Survey Prevalence		Catalonia Population Survey Prevalence		Catalonia SIDIAP Insomnia Diagnostics	
Age Group	Total	>55 y.	Total	>55 y.	Total	>55 y.
Insomnia Symptoms	43.4%	43.3%	41.39%	45.2%	N/A	N/A
Chronic Insomnia Syndrome	13.7%	14.5%	12.9%	14.2%	N/A	N/A
Chronic Insomnia Disorder	14%	17.9%	13.6%	18.2%	5.1%	18.5%

Note: The SIDIAP does not record symptom- or syndrome-level insomnia data.

**Table 2:** Prevalence of chronic insomnia disorder by sex in population surveys and SIDIAP data.

	Spain Population Survey Prevalence		Catalonia Population Survey Prevalence		Catalonia SIDIAP Insomnia Diagnostics	
Sex	Women	Men	Women	Men	Women	Men
Insomnia Symptoms	N/A	N/A	N/A	N/A	N/A	N/A
Chronic Insomnia Syndrome	N/A	N/A	N/A	N/A	N/A	N/A
Chronic Insomnia Disorder	16.4%	14.6%	17.1%	13.8%	3.4%	1.9%

Note: SIDIAP includes only ICD-10 coded insomnia diagnoses.

## Data Availability

In accordance with current European and national laws, the data used in our study were only available to the researchers participating in our study. Thus, we were not allowed to distribute publicly available data to other parties. Researchers from public institutions can request data from SIDIAP if they comply with certain requirements. Further information is available online (https://www.sidiap.org/index.php/en/solicituds-en, accessed on 25 November 2025).

## Abbreviations

The following abbreviations are used in this manuscript.

ICSD-3	International Classification of Sleep Disorders (3rd version)
EPINSOM	Epidemiology Study of Insomnia in Spain
